# The association of dietary and lifestyle indices for insulin resistance with the risk of cardiometabolic diseases among Iranian adults

**DOI:** 10.1038/s41598-023-33505-4

**Published:** 2023-04-17

**Authors:** Farshad Teymoori, Mitra Kazemi Jahromi, Hamid Ahmadirad, Ghazal Daftari, Ebrahim Mokhtari, Hossein Farhadnejad, Parvin Mirmiran, Fereidoun Azizi

**Affiliations:** 1 Nutrition and Endocrine Research Center, Research Institute for Endocrine Sciences, Shahid Beheshti University of Medical Sciences, Tehran, Iran; 2 Department of Nutrition, School of Public Health, Iran University of Medical Sciences, Tehran, Iran; 3 Endocrinology and Metabolism Research Center, Hormozgan University of Medical Sciences, Bandar Abbas, Iran; 4 School of Medicine, Tehran University of Medical Science, Tehran, Iran; 5 Endocrine Research Center, Research Institute for Endocrine Sciences, Shahid Beheshti University of Medical Sciences, Tehran, Iran

**Keywords:** Endocrine system and metabolic diseases, Metabolic disorders, Endocrinology

## Abstract

The current study aimed to examine the possible association of the dietary index for insulin resistance (DIR) and the lifestyle index for insulin resistance (LIR), determined by dietary components, body mass index, and physical activity, with the risk of cardiometabolic diseases, including insulin resistance (IR), hypertension (HTN), diabetes, and metabolic syndrome (MetS). This prospective cohort study was performed on 2717 individuals aged ≥ 19 years in the framework of the Tehran Lipid-Glucose Study. Data on nutritional intakes were assessed using a validated food frequency questionnaire. Logistic regression models were used to determine the odds ratio and 95% confidence intervals (ORs and 95% CIs) of cardiometabolic diseases across tertiles of DIR and LIR scores. During 3-years of follow-up, the incidence rate of diabetes, IR, HTN, and MetS was 3%, 13%, 13.9%, and 17%, respectively. In the multi-variables model, after controlling all potential confounders, the risk of IR(OR: 1.65, 95% CI 1.01–2.69, P-trend = 0.047), diabetes (OR: 1.95, 95% CI 1.02–3.74, P-trend = 0.058), and HTN(OR: 1.52, 95% CI 1.07–2.15, P-trend = 0.016) was increased across tertiles of DIR score. Also, the risk of IR (OR: 2.85, 95% CI 1.72–4.73, P-trend < 0.001), diabetes(OR: 2.44, 95% CI 1.24–4.78, P-trend = 0.004), HTN(OR: 1.95, 95% CI 1.35–2.81, P-trend < 0.001), and MetS (OR: 2.87, 95% CI 1.96–4.18, P-trend < 0.001) were increased across tertiles of LIR score. Our findings reported that a dietary pattern with a higher DIR score and a lifestyle with a higher LIR score might be related to increased cardiometabolic disorders, including diabetes, HTN, Mets, and IR in Iranian adults.

## Introduction

Insulin is an essential hormone that affects different organs such as adipose tissue, liver, muscles, brain, kidneys, vasculature, etc.^1,2^. Insulin-related disorders, including insulin resistance (IR) and hyperinsulinemia, are considered early predictors of metabolic disorders^3,4^. Chronic IR can result in chronic diseases, such as Type 2 diabetes (T2D), metabolic syndrome (MetS), hypertension (HTN), and cardiovascular disease^5,6^. The latest estimates on the prevalence of MetS, T2D, and HTN in the Iranian population are about 40%^7^, 13.2%^8^, and 25%, respectively^9^.

Lifestyle and dietary habits are known as the most important modifiable determinants of IR^10^. Several studies recently focused on the possible role of dietary insulin index and insulin load in the prediction of cardiometabolic risk factors, such as hyperglycemia, dyslipidemia, IR, and obesity, which indicates interesting findings^11–14^. Also, previously, Tabung and his colleagues developed indices to determine the insulinemic potential of diet and lifestyle, including the empirical dietary index for IR (EDIR) and empirical lifestyle index for IR (ELIR) for predicting IR using the dietary and lifestyle factors among the U.S population^15^; the results of some recent studies have shown that the dietary pattern and lifestyle with a high score of EDIR and ELIR may play a risk factor role in the etiology of chronic diseases such as IR^16^, type 2 diabetes^17,18^, fatty liver diseases^19^, and cardiovascular outcomes^20^. Furthermore, recently we developed and validated two indices to determine the potential of diet and lifestyle factors for predicting IR among Iranian adults^21^. The dietary index for IR (DIR) was defined according to the dietary items with a significant relationship with IR. Also, the lifestyle index for IR (LIR) was developed based on the body mass index (BMI) and physical activity (PA), besides some related dietary items with IR^21^. A higher score of DIR and LIR indices shows a higher potential of diet and lifestyle factors to increase the risk of insulin resistance and vice versa.

Although some lifestyle indices have been applied to a variety of chronic diseases, as DIR and LIR were recently proposed, their strengths in predicting the risk of Chronic diseases have not been investigated previously. The present study aims to assess the association of DIR and LIR, developed in the Iranian population, with the risk of incidence of cardiometabolic diseases including IR, HTN, diabetes, and metabolic syndrome (MetS) in the framework of the Tehran Lipid-Glucose Study (TLGS).

## Materials and methods

### Study participants

The TLGS study was started in 1999 in Tehran city, and its data are collected prospectively at 3-year intervals. Detailed information is provided elsewhere^22^. The current study was performed within the TLGS framework.

**Figure 1:**
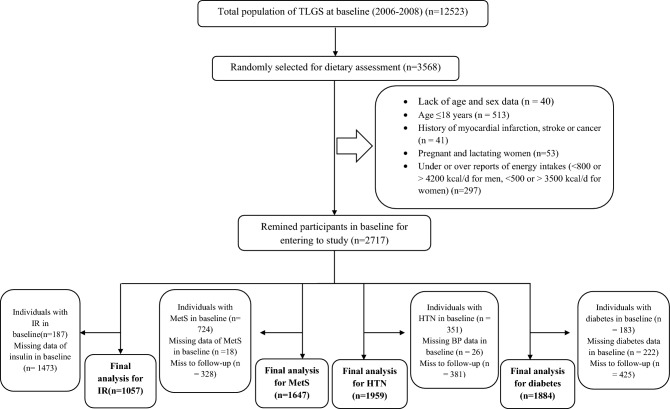
Flowchart of the study population and follow-up.

In the third survey of the TLGS (2006–2008), out of 12523 participants, 3568 were randomly selected for dietary evaluation. We excluded individuals based on at least one of the following criteria: lack of age and sex data (n = 40), age ≤18 years (n = 513), history of myocardial infarction, stroke, or cancer (n = 27 men and 14 women), daily energy intake less than 800 or more than 4200 kcal per day in men and out of the range of 500-3500 kcal per day in women (n = 297) as well as pregnant or lactating women (n = 53). It is noteworthy that some people fell into more than one category. Finally, 2717 (45.6) participants in the third survey (baseline) remained to enter the study for follow-up until the fourth survey (end of follow-up, 2009–2011) (Fig. 1). It should be mentioned that only 1244 participants of them had insulin data.

After excluding diabetic patients in baseline (n = 183) and people with missing diabetes data in baseline (n = 222) and follow-up (n = 425), 1884 participants remained for final analysis on diabetes. For analyses on HTN, after excluding participants with HTN in baseline (n = 351) and those with missing BP data in baseline (n = 26) and follow-up (n = 381), 1959 participants remained.

For analyses on MetS, individuals with MetS in baseline (n = 724) and those with missing data of MetS in baseline (n = 18) and follow-up (n = 328) were excluded, and finally, 1647 individuals remained for final analysis. Also, from 1244 participants with insulin data, after excluding individuals with IR (n = 187) in baseline, 1057 individuals were followed up until the fourth survey (follow-up rate = 100%), and all of them remained for the final analysis on IR.

### Demographic and anthropometric assessments

Demographic information was assessed using a pretested questionnaire at baseline, in which subjects were asked to answer questions on age, sex, smoking status, educational level, medical history, medication use, etc.

Anthropometric assessments were performed by a skilled and experienced dietitian at baseline. Weight was measured using a SECA digital weighing scale (Seca 707; Seca Corporation, Hanover, Maryland) with an accuracy of up to 100 g over light clothing. Height was measured using a stadiometer with a minimum of 1 cm in a standing position without shoes and shoulders in normal alignment. BMI was computed as weight (Kg) divided by the square of height (m^2^). Waist circumference (WC) was measured with an accuracy of 0.1 cm using a non-elastic tape meter, between the lowest chest ribs and the iliac crest at the umbilicus level, over light clothing, and without any pressure.

### Biochemical and clinical measurements

Biochemical and clinical measurements were performed at baseline or the end of the follow-up of the study. Blood samples of all participants were collected after 12–14 h of overnight fasting in a steady-state sitting position between 7:00 and 9.00 AM and centrifuged within 30–45 min of collection. All samples were analyzed at the TLGS research laboratory on collection using Selectra 2 auto-analyzer (Vital Scientific, Spankeren, Netherlands). Fasting blood sugar (FBS) was measured using an enzymatic colorimetric method with glucose oxidase. Inter/intra-assay coefficient variations for FBS were both 2.2% for FBS. The 2-h oral glucose tolerance test was performed using 82.5 g of glucose monohydrate solution (equivalent to 75 g anhydrous glucose), which was administered orally to all individuals aged > 20 years, except diabetic patients on anti-diabetic drug therapy based on the prescription of the endocrinologist. Fasting serum insulin (FSI) was measured via electrochemiluminescence immunoassay (ECLIA), using Roche Diagnostics kits and Roche/Hitachi Cobas e-411 analyzer (Gmbh, manhim, Germany). Inter- and intra-assay coefficient variations for insulin were 1.2 and 3.5, respectively. Triglycerides (TGs) levels were measured using the enzymatic colorimetric method with glycerol phosphate oxidase. Inter- and intra-assay CVs for TGs were 0.6 and 1.6%, respectively. High-density lipoprotein cholesterol (HDL-C) of serum was measured after precipitation of the apolipoprotein B-containing lipoproteins with phosphotungstic acid (PTA).

Blood pressure (BP) was measured after resting for at least 15 minutes sitting on a chair, twice on the right arm, with a minimum interval of 30 seconds, using a mercury sphygmomanometer and Korotkoff sound technique, with an accuracy of 2 mmHg. The average of the two measurements was considered the subject’s BP; systolic BP (SBP) with the first sound to be heard and diastolic BP (DBP) with the disappearance of the sound was recorded.

### Definitions

#### IR

Homeostatic Model Assessment of Insulin Resistance (HOMA-IR) was used to assess IR (HOMA-IR = FBS (mmol/L) × Insulin (μU/mL)/22.5). HOMA-IR ≥ 3.2 was determined as the criteria for IR^23^.

#### Diabetes

Diabetes was defined based on the criteria of the American Diabetes Association (ADA) as FPG ≥ 126 mg/dl or 2-h post 75 g glucose load ≥ 200 mg/dl or taking oral hypoglycemic medication^24^.

#### HTN

SBP ≥ 140, DBP ≥ 90, or taking antihypertensive medications was determined as criteria for HTN^25^.

#### MetS

MetS was defined according to the joint interim statement as the presence of any 3 of 5 following factors^26^: (1) abdominal obesity as WC ≥ 95 cm for both genders, according to the new cutoff points of WC for Iranian Adults^27^; (2) FPG ≥ 100 mg/dl or drug treatment; (3) fasting TGs ≥ 150 mg/dl or drug treatment; (4) fasting HDL-C < 50 mg/dl for women and < 40 mg/dl for men or drug treatment; and (5) high BP was defined as SBP ≥ 130 mmHg, DBP ≥ 85 mmHg, or antihypertensive drug treatment.

#### Visceral adiposity index (VAI)

This index was calculated for men and women as below:

Males: VAI=(WC (cm)/(39.68+(1.88×BMI (kg/m2)))×(TG (mmol/l)/1.03)×(1.31/HDL (mmol/l)).

Females: VAI = (WC (cm)/(39.58+(1.89×BMI (kg/m2)))×(TG (mmol/l)/0.81)×(1.52/HDL (mmol/l)).

#### Waist residual BMI

We regressed WC on BMI to obtain BMI-independent WC values, calculated as differences between each individual’s WC and the WC predicted by BMI.

### Physical activity assessment

The modified and validated version of the Modified Activity Questionnaire (MAQ) for the Iranian population was used to assess participants’ PA status at baseline^28^. Individuals were asked to report the frequency and time spent for PA during the past year as light, moderate, hard, and very hard intensity. PA levels were converted to metabolic equivalent hours per week (MET.h/Wk.). Detailed information is available elsewhere^16^.

### Dietary intake assessment

Dietary intakes were assessed using a valid and reliable 168-item semi-quantitative food frequency questionnaire (FFQ). The FFQ validity was previously fulfilled by comparing food groups values derived from the questionnaire with values estimated by twelve 24-h dietary recall surveys^29,30^. The frequency of consumption for each food item during the past year on a daily, weekly, or monthly basis was collected during a face-to-face interview by trained and skilled dieticians. Portion sizes of consumed foods reported in domestic measures were then transformed into a gram scale using the United States Department of Agriculture (USDA) food composition table (FCT). Also, USDA FCT is used to compute energy and nutrient content. The Iranian FCT was also used for some local food items unavailable in USDA FCT. Dietary intakes in the third survey (2008-2011) of TLGS were considered as exposure at baseline.

### Calculation of indices

The DIR and LIR scores were calculated using the method explained previously^21^. The DIR index encompasses 12 dietary items, including pickles, refined grains, doogh, lemon juice, sweetened beverages, fish (as items directly related to IR), starchy vegetables, snacks, low-fat dairy, broth, red meat, and high-fat dairy (as items inversely related to IR). Also, the LIR index contained seven dietary and lifestyle items, including BMI, refined grain, doogh (as items directly related to IR), low-fat dairy, physical activity, starchy vegetables, and high-fat dairy (as items inversely related to IR). Dietary intakes of each food group were converted into serving sizes per 1000 Kcal of energy intake. Then each component, including dietary items, BMI, and physical activity (MET.h/wk.), multiplied by their weights, which were reported in the development study^21^, and values for all components of each DIR or LIR were summed to achieve their final score. A higher score of DIR and LIR indices means a higher potential for diet and lifestyle factors to increase the risk of insulin resistance and vice versa.

### Statistical analysis

Data were analyzed using the Statistical Package for Social Sciences (version 20.0; SPSS Inc, Chicago, IL). Histogram charts and Kolmogorov–Smirnov analysis were used to assess the normality of variables. Participants were categorized according to DIR and LIR tertiles. Baseline characteristics of individuals were expressed for continuous and categorical variables as mean ± standard deviation (SD) or median (25–75) interquartile range (IQR) and percentage, respectively. Trends of qualitative and quantitative variables across tertiles of DIR (as the median value in each tertile) were tested using Chi-square and linear regression. Multivariable logistic regression was used to estimate the risk of IR, T2D, HTN, and MetS as dependent variables, and the DIR and LIR scores as independent variables; odds ratio (OR) and 95% confidence interval (CI) were reported. The regression models were adjusted for age, sex, energy intake, smoking, education level, and occupation status, in addition to PA and VAI (only for DIR) and TGs to HDL-C ratio, and waist residual BMI (only for LIR). P-values < 0.05 were considered statistically significant.

### Ethics approval and consent to participate

Informed written consent was obtained from participants. All procedures performed in studies involving human participants adhered to the ethical standards of the institutional and/or national research committee and with the 1964 Helsinki declaration and its later amendments or comparable ethical standards. The study protocol was approved by the research council of the Research Institute for Endocrine Sciences, Shahid Beheshti University of Medical Sciences

## Results

The mean (SD) age and BMI of participants (45.6 male) were 39.9 (14.4) years and 26.8(4.9) kg/m2, respectively. After nearly three years of follow-up, respectively, 3%, 13%, 13.9% and 17% of D2M, IR, HTN, and MetS occurred.

General characteristics and clinical measurements of participants based on the tertile of DIR are indicated in Table 1. Education status and HDL-C decreased across tertiles of DIR, however, physical activity, BMI, WC, FSI, SBP, HOMA-IR, TGs, TG: HDL, and VAI increased. There was no significant difference in smoking, occupation status, and fasting blood sugar across tertiles of DIR.

**Table 1:** Baseline characteristics across tertiles of the DIR among 2717 participants of the third examination of the Tehran Lipid and Glucose Study (2006–2008).

	T1 (n = 906)	T2 (n = 905)	T3 (n = 906)	P_trend^†^
Age (y)	41.2 ± 14.9	38.9 ± 13.9	39.6 ± 14.4	0.025
Male (%)	40.9	45.7	50.2	< 0.001
Current smokers (%)	11.8	13.0	12.9	0.483
Physical activity (MET-h/wk)	75.5 ± 54.0	81.5 ± 56.0	82.5 ± 57.9	0.009
BMI (kg/m^2^)	26.2 ± 4.5	26.8 ± 5.1	27.5 ± 5.0	< 0.001
Waist circumference (cm)	87.5 ± 12.8	88.8 ± 13.5	91.41 ± 13.9	< 0.001
Education status (≥ Diploma), (%)	27.1	27.6	20.2	0.001
Occupation status (employed), (%)	81.4	80.9	80.3	0.540
FSI (mU/mL)	8.34 ± 5.24	8.86 ± 4.83	10.39 ± 6.76	< 0.001
FBS (mg/dl)	91.1 ± 22.5	90.6 ± 21.8	93.1 ± 26.1	0.062
SBP (mmHg)	110.7 ± 17.3	111.4 ± 15.6	113.7 ± 17.3	< 0.001
HOMA-IR	1.58 (1.06–2.30)	1.67 (1.19–2.42)	1.97 (1.36–3.06)	< 0.001
TGs (mmol/l)	1.51 ± 1.01	1.54 ± 0.95	1.75 ± 1.09	< 0.001
HDL-c (mmol/l)	1.12 ± 0.26	1.10 ± 0.26	1.06 ± 0.25	< 0.001
TG: HDL	1.52 ± 1.36	1.56 ± 1.20	1.85 ± 1.47	< 0.001
VAI	1.86 (1.15–2.90)	1.92(1.21–3.09)	2.17(1.34–3.65)	< 0.001
DIR	− 0.03 (− 0.08–0.02)	0.07 (0.02–0.13)	0.17 (0.10–0.25)	
LIR	8.22 (5.35–10.86)	8.10 (5.26–10.79)	8.37 (5.59–11.65)	

Data are presented as mean ± SD or median (IQR) for continuous, and percent and categorical variables.

*BMI* body mass index, *FSI* fasting serum insulin, *FBS* fasting blood sugar, *SBP* systolic blood pressure, *HOMA-IR* the homeostasis model assessment-estimated insulin resistance, *TGs* triglycerides, *HDL-c* high-density lipoprotein cholesterol, *VAI* visceral adiposity index.

^†^P for trend was calculated using linear regression and chi-square test for continuous and categorical variables, respectively.

Table 2 shows the intake of food groups and nutrients according to the tertile of the DIR. Across tertiles of DIR, the starchy vegetables, snacks, low-fat dairy, broth, red meat, high-fat dairy, total fat, and total protein decreased, however, dietary intake of pickles, refined grains, doogh, lemon juice, sweetened beverages, fish, and carbohydrates increased. There were no significant differences in other variables across the tertile of DIR.

**Table 2:** Baseline dietary intakes across tertiles of the DIR among 2717 participants of the third examination of the Tehran Lipid and Glucose Study (2006–2008).

Variables	T1 (n = 906)	T2 (n = 905)	T3 (n = 906)	P_trend^†^
Food groups				
Pickles (Srv/d)	0.34 (0.20–0.64)	0.45 (0.24–0.70)	0.64 (0.30–0.87)	< 0.001
Refined grains (Srv /d)	3.74 ± 2.14	5.14 ± 2.88	6.73 ± 4.60	< 0.001
Doogh (Srv/ wk)	0.53 (0.18–1.06)	0.79 (0.30–1.59)	1.06 (0.37 -2.98)	< 0.001
Lemon juice (Srv/ wk)	0.50 (0.15–1.00)	0.66 (0.33–1.86)	0.66 (0.33–2.33)	< 0.001
Sweetened beverages (Srv/ wk)	0.54 (0.08–1.17)	0.61 (0.26–2.33)	0.81 (0.30–2.61)	< 0.001
Fish (Srv/d)	0.21 (0.11–0.37)	0.23 (0.12–0.47)	0.23 (0.11–0.52)	< 0.001
Starchy vegetables (Srv/d)	0.21 (0.12–0.39)	0.19 (0.12–0.33)	0.16 (0.09–0.27)	< 0.001
Snacks (Srv/d)	3.11 ± 2.50	2.48 ± 1.72	1.92 ± 1.49	< 0.001
Low-fat dairy (Srv/d)	1.00 (0.35–1.33)	0.71 (0.25–1.25)	0.44 (0.17–1.25)	< 0.001
Broth (Srv/wk)	0.01(0.00–0.04)	0.01(0.00–0.03)	0.01(0.00–0.02)	< 0.001
Red meat (Srv/d)	0.54 (0.27–1.02)	0.43 (0.23–0.77)	0.33 (0.16–0.59)	< 0.001
High-fat dairy (Srv/d)	1.77 ± 1.19	1.31 ± 0.83	0.99 ± 0.76	< 0.001
Nutrient intakes				
Energy intake, Kcal	2202 ± 671	2244 ± 677	2192 ± 739	0.712
Carbohydrates (% of energy)	55.6 ± 7.0	57.6 ± 6.8	59.5 ± 7.1	< 0.001
Total fat (% of energy)	32.9 ± 6.6	31.4 ± 6.7	29.4 ± 7.1	< 0.001
Total protein (% of energy)	14.1 ± 2.4	13.6 ± 2.3	13.3 ± 2.4	< 0.001

Data are presented as mean ± SD or median (IQR).

^†^P for trend was calculated using linear regression and chi-square test for continuous and categorical variables, respectively.

Table 3 demonstrates the relationship between DIR and LIR scores with the incidence of IR and MetS. Based on all logistic models, participants with higher scores of DIR have a higher risk of IR incidence. The OR (95% CI) of IR for highest vs. lowest tertiles of DIR were 1.65(1.01–2.69), and P for trend = 0.047 in the fully adjusted model. Although the higher DIR score was significantly associated with a higher risk of MetS based on logistic models 1 and 2, after adjusting the VAI in the final model, this association become non-significant (OR: 1.32; (95% CI 0.94–1.87), P-trend=0.091). In the final model adjusted for confounding variables, the OR (95% CI) of IR and MetS for participants who were in the highest vs. lowest tertiles of LIR were 2.85(1.72–4.73) and 2.87(1.96–4.18), respectively (P for trend < 0.001).

**Table 3:** The association between the insulinemic dietary and lifestyle indices with the incidence of insulin resistance and metabolic syndrome: the Tehran Lipid and Glucose Study.

	Insulin resistance				Metabolic syndrome			
	Tertiles of indices			P trend	Tertiles of indices			P trend
	T1	T2	T3		T1	T2	T3	
DIR								
Median score	− 0.04	0.05	0.18		− 0.04	0.05	0.18	
Case/Total	38/353	46/351	54/353		93/549	89/549	112/549	
Model 1*	1.00 (Ref)	1.40 (0.86–2.28)	1.74 (1.08–2.80)	0.023	1.00 (Ref)	1.05 (0.75–1.47)	1.41 (1.02–1.95)	0.030
Model 2^†^	1.00 (Ref)	1.38 (0.85–2.26)	1.71 (1.06–2.76)	0.028	1.00 (Ref)	1.05 (0.75–1.47)	1.41 (1.01–1.95)	0.032
Model 3^‡^	1.00 (Ref)	1.34 (0.81–2.21)	1.65 (1.01–2.69)	0.047	1.00 (Ref)	1.03 (0.72–1.47)	1.32 (0.94–1.87)	0.091
LIR								
Median score	7.60	10.81	13.37		3.45	7.67	11.35	
Case/Total	26/349	38/350	73/349		57/543	86/545	150/543	
Model 1*	1.00 (Ref)	1.48 (0.86–2.54)	3.23 (1.97–5.30)	< 0.001	1.00 (Ref)	1.36 (0.94–1.98)	2.76 (1.93–3.94)	< 0.001
Model 2^†^	1.00 (Ref)	1.51 (0.88–2.59)	3.20 (1.95–5.25)	< 0.001	1.00 (Ref)	1.39 (0.95–2.02)	2.81 (1.96–4.02)	< 0.001
Model 3^‡^	1.00 (Ref)	1.38 (0.79–2.40)	2.85 (1.72–4.73)	< 0.001	1.00 (Ref)	1.15 (0.77–1.71)	2.87 (1.96–4.18)	< 0.001

*Model 1: adjusted for age and sex.

^†^Model 2: adjusted for model 1 and energy intake, smoking, education level, occupation status, and physical activity (only for DIR).

^‡^Model 3: adjusted for model 2 and baseline VAI (for DIR) and TG to HDL-c ratio and waist residual BMI (for LIR).

The association between DIR and LIR scores with the risk of HTN and diabetes has shown in Table 4. In the final model, subjects who were in the third versus those in the first tertile of DIR had a higher risk of HTN (OR: 1.52; 95% CI 1.07–2.15), P-trend=0.016) and diabetes (OR: 1.95; 95% CI 1.02–3.74), P-trend=0.058). Also, based on the final model, the OR (95% CI) of HTN and diabetes for participants who were in the highest vs. lowest tertiles of LIR were 1.95 (1.35–2.81), P-trend < 0.001 and 2.44 (1.24–4.78), P for trend = 0.004, respectively.

**Table 4:** The association between the insulinemic dietary and lifestyle indices with the incidence of hypertension and diabetes: the Tehran Lipid and Glucose Study.

	Hypertension				Diabetes			
	Tertiles of indices			P trend	Tertiles of indices			P trend
	T1	T2	T3		T1	T2	T3	
DIR								
Median score	− 0.02	0.07	0.16		− 0.03	0.07	0.16	
Case/Total	82/652	83/653	108/654		17/628	28/628	29/628	
Model 1*	1.00 (Ref)	1.21 (0.85–1.72)	1.71 (1.22–2.40)	0.002	1.00 (Ref)	2.27 (1.19–4.31)	2.09 (1.10–3.95)	0.030
Model 2^†^	1.00 (Ref)	1.22 (0.85–1.74)	1.67 (1.19–2.35)	0.003	1.00 (Ref)	2.38 (1.25–4.55)	2.14 (1.12–4.08)	0.026
Model 3^‡^	1.00 (Ref)	1.17 (0.82–1.68)	1.52 (1.07–2.15)	0.016	1.00 (Ref)	2.34 (1.22–4.47)	1.95 (1.02–3.74)	0.058
LIR								
Median score	3.77	7.98	12.01		4.19	8.28	12.42	
Case/Total	55/646	71/648	146/647		13/622	19/623	42/624	
Model 1*	1.00 (Ref)	0.96 (0.65–1.41)	2.07 (1.44–2.96)	< 0.001	1.00 (Ref)	1.18(0.57–2.46)	2.47 (1.27–4.79)	0.003
Model 2^†^	1.00 (Ref)	0.95 (0.64–1.41)	2.02 (1.41–2.91)	< 0.001	1.00 (Ref)	1.22 (0.58–2.54)	2.53 (1.30–4.95)	0.003
Model 3^‡^	1.00 (Ref)	0.92 (0.62–1.37)	1.95 (1.35–2.81)	< 0.001	1.00 (Ref)	1.20 (0.57–2.50)	2.44 (1.24–4.78)	0.004

*Model 1: adjusted for age and sex.

^†^Model 2: adjusted for model 1 and energy intake, smoking, education level, occupation status, and physical activity (only for DIR).

^‡^Model 3: adjusted for model 2 and baseline VAI (for DIR) and TG to HDL-c ratio and waist residual BMI (for LIR).

## Discussion

The present population-based cohort study showed that higher scores of DIR and LIR were significantly associated with the increased risk of cardiometabolic diseases, including IR, HTN, diabetes, and MetS, independent of confounding factors in the Iranian adult population.

The investigation of the possible association of dietary patterns alone or combined with other environmental factors in various aspects with the risk of various cardiometabolic disorders is becoming one of the most important aspects of epidemiological investigations because it is reported that this complex involves the interrelationships between different key factor and so can create a wide insight into this regard. Since IR has been recognized as an early metabolic dysfunction stage previously for various chronic diseases such as type 2 diabetes, HTN, cardiovascular diseases, and MetS^31–33^; therefore, investigating the insulinemic effect of diet in combination with other factors related to lifestyle in the prediction of the above-mentioned chronic diseases by influencing the risk of IR can help identify new aspects of the role of diet and lifestyle in the risk of chronic diseases.

To the best of our knowledge, for the first time, this study has investigated the relationship between indices related to dietary insulin potential and lifestyle (DIR and LIR), which have been determined and validated in the Iranian population^21^, with the risk of cardiometabolic disorders in adults. However, our findings are comparable with the results of previous studies that assessed the association of dietary insulin indices and risk of cardiometabolic abnormalities, including dyslipidemia, hyperglycemia, IR, and adiposity^11–14^. Nimptsch et al. suggested that a diet with higher insulin index is positively related to low HDL-C and hypertriglyceridemia; but, no association was observed between dietary insulin indices and low-density lipoprotein-cholesterol and glycemic indices^11^. Also, in a cohort study, a direct association was found between the high insulinemic diet and the risk of IR^14^. In a cross-sectional study, high insulin index diet was related to a higher risk of obesity among women^13^. Furthermore, Mozaffari et al. indicated that adherence to a high dietary insulin load diet is positively associated with blood glucose level, however, no significant findings were observed for adiposity and dyslipidemia^12^.

Also, the results of our study are in line with the findings of previous studies that focused on the association of EDIR and ELIR, developed as dietary and lifestyle patterns for prediction of IR by Tabung et al. study^15^, with the risk of various chronic diseases^17,19,20,34^. Farhadnejad et al. study reported that a lifestyle with a higher score of ELIR and a diet with a higher EDIR score may be related to an increased risk of type 2 diabetes^17^. Also, another prospective study on the Iranian population showed that a lifestyle and diet with a higher score of EDIR and ELIR are remarkably linked to a higher risk of cardiovascular disease and coronary heart disease outcomes in the adult population^20^. Furthermore, two observational studies revealed that higher adherence to a dietary pattern with a higher score of EDIR can be a major risk factor for the development of hepatic steatosis and fibrosis and the increased risk of non-alcoholic fatty liver disease^19,34^. Similar to the results of previous studies, the results of our study strongly support the hypothesis that a diet and lifestyle with high insulin potential can increase the risk of cardiometabolic disorders. It is worth mentioning that our study had three important and major differences compared to the previous study, which multiplies the importance of the findings of our study; in this study, we used insulin indices that were previously designed and validated for our study population. Also, we investigated several different cardiometabolic disorders including type 2 diabetes, MetS, HTN, and IR in the same population.

It is still not possible to clearly and fully identify the reasons or characteristics explaining the role of the insulinemic potential of diet and lifestyle in the pathogenesis of metabolic disorders. But since IR is an intermediate and underlying factor for metabolic disorders such as type 2 diabetes, MetS, and HTN, a diet or lifestyle with high insulinemic properties, is determined based on the intake of different food components in combination with lower levels of physical activity and higher body mass index, with an effect on the risk of hyperinsulinemia and IR in the long term^17^, plays an important role in increasing the risk of above-mentioned cardiometabolic disorders^31–33^. In our study, a dietary pattern with a higher score of DIR, which was characterized by higher consumption of refined grains and sweetened beverages and lower consumption of starchy vegetables and low-fat dairy products, may have an adverse effect on insulin secretion and insulin function, and it can increase the risk of IR-related metabolic diseases^17,35–37^. Consequently, an increased risk of IR in individuals with a higher score of DIR, along with increased inflammation and increment oxidative stress, may increase the development of cardiometabolic disorders such as dyslipidemia, hyperglycemia, and HTN^38–40^.

In addition to food components, a high BMI level is another important determinant of the LIR index, which can play an important role in the initiation and progression of IR and related disorders. It has been suggested that elevated BMI and central obesity are independently associated with increased risk of IR and various metabolic disorders^41^; therefore, the main part of the insulinemic potential of LIR in the prediction of the risk of metabolic diseases, including type 2 diabetes, MetS, and HTN was related to a higher level of BMI in individuals. Also, physical activity is another important determinant of LIR that can play a remarkable role in the pathogenesis of IR and its- related disorders^42^. Individuals with a sedentary lifestyle and physical inactivity can potentially be more prone to the occurrence of IR and insulin-related diseases such as type 2 diabetes, MetS, and HTN because the lower level of physical activity is potentially linked to increased inflammation, increased insulin insensitivity, improper effect on body weight and increase adiposity, and decrease the use of energy in various body organs, so it consequently can increase insulin metabolism-related disorders^42–44^. Finally, based on Tables 3 and 4, like previous epidemiological studies, our study showed that the collective contributions of the insulinemic dietary pattern alongside a high BMI level and sedentary lifestyle in the form of LIR can remarkably be a stronger risk factor of the increased risk of IR and its related metabolic diseases such as type 2 diabetes, HTN, and MetS compared to DIR.

In the current study, we have determined the insulinemic potential of diet and lifestyle by DIR and LIR indices that can be predictive indices for the concentrations of the insulin response biomarkers and HDL-C to TGs ratio without the need to measure serum insulin, TGs, or HDL-C levels in individuals. Also, calculating the DIR and LIR scores helps us to predict the role of lifestyle and diet in predicting the risk of chronic diseases, including cardiometabolic disorders, by determining their influence on the mediating role of IR in metabolic disorders. It should be noted that, although the insulinemic potential of dietary and lifestyle indices was previously determined by EDIR and ELIR^15^, and their possible relationship with chronic diseases had been investigated in various studies, considering that the pattern of chronic disease incidents as well as dietary habits and lifestyle of individuals in the Middle East and North Africa (MENA) region, such as Iran, may have differences with Western countries, therefore, we determined the insulinemic potential of dietary and lifestyle using two indices (LIR and DIR) that developed and validated for the Iranian population^21^; using these insulinemic indices in our study help to predict the role of lifestyle and diet on IR and subsequently their impact on the occurrence of various chronic diseases, including cardiometabolic disorders in Iran and other communities in the MENA region.

Our study had several strengths, including its prospective design, the appropriate sample size to explore the relationship between our exposures (LIR and DIR) and desired outcomes, and a 100% follow-up rate. Also, the present study is the first investigation that assessed the possible role of the Iranian indices of the dietary and lifestyle insulinemic potential on the risk of various metabolic disorders, including type 2 diabetes, MetS, HTN, and IR. Furthermore, in the current study, we used valid and reliable questionnaires to collect dietary intake data. However, the current study had some limitations that deserve to mention. First, because the Iranian FCT is not complete in some food items and micronutrients, we used the USDA FCT to calculate participants’ energy and nutrient intakes from their diet. Second, using the FFQ to collect the participants’ dietary information makes measurement errors inevitable; however, similar to most epidemiological studies, we used a validated questionnaire to minimize measurement errors. Also, we cannot precisely estimate alcohol consumption as the main risk factor for metabolic disorders in our country because alcohol consumption is forbidden due to cultural and religious beliefs; therefore, we cannot adjust the confounding effect of alcohol intake in the final analysis. The use of indirect measurement for PA is another limitation that cannot be completely ignored, even though direct measurement of PA is uncommon in large population studies. However, to improve assessment precision, we collected the PA data using a valid and reliable questionnaire during a face-to-face interview. Finally, despite adjusting the effects of various confounders in this study, residual confounding due to unknown or unmeasured confounders like genetic background or psychological stress cannot confidently be excluded.

## Conclusions

Our findings revealed that higher LIR and DIR scores may be related to an increased risk of metabolic disorders, including type 2 diabetes, HTN, IR, and MetS in the Iranian adult population. It suggested that more prospective studies are performed to examine the possible role of the high insulinemic potential of lifestyle and dietary patterns in predicting the risk of metabolic disorders in other populations.

## Data Availability

The datasets analyzed in the current study are available from the corresponding author on reasonable request.

## Abbreviations

ADA: American diabetes association
BMI: Body mass index
BP: Blood pressure
CI: Confidence interval
DIR: Dietary index for IR
ECLIA: Electro-chemiluminescence immunoassay
FBS: Fasting blood sugar
FCT: Food composition table
FFQ: Food frequency questionnaire
HDL-C: High-density lipoprotein cholesterol
HOMA-IR: Homeostatic model assessment of insulin resistance
IR: Insulin resistance
IQR: Interquartile range
LIR: Lifestyle index for IR
MAQ: Modified activity questionnaire
MET: Metabolic equivalent
MetS: Metabolic syndrome
OR: Odds ratio
PA: Physical activity
SBP: Systolic blood pressure
SD: Standard deviation
SPSS: Statistical package for social sciences
TGs: Triglycerides
TLGS: Tehran lipid-glucose study
USDA: United States department of agriculture
VAI: Visceral adiposity index
WC: Waist circumference
